# Hemispheric division of function is the result of independent probabilistic biases

**DOI:** 10.1016/j.neuropsychologia.2009.03.005

**Published:** 2009-07

**Authors:** Andrew J.O. Whitehouse, Dorothy V.M. Bishop

**Affiliations:** a Telethon Institute for Child Health Research, Centre for Child Health Research, The University of Western Australia, PO Box 855, West Perth, Western Australia 6872, Australia; b Department of Experimental Psychology, University of Oxford, South Parks Road, Oxford OX1 3UD, United Kingdom

**Keywords:** Cerebral lateralisation, Language, Visuospatial, Functional transcranial Doppler ultrasonography

## Abstract

Verbal and visuospatial abilities are typically subserved by different cerebral hemispheres: the left hemisphere for the former and the right hemisphere for the latter. However little is known of the origin of this division of function. Causal theories propose that functional asymmetry is an obligatory pattern of organisation, while statistical theories maintain this is a reflection of independent, probalistic biases. The current study investigated lateralisation for language production and spatial memory using functional Transcranial Doppler in 75 healthy adults (45 right handed, 27 left-handed, 3 ambidextrous). The majority of participants had language abilities lateralised to the left-hemisphere and spatial memory to the right hemisphere, while around one-quarter of participants had these functions lateralised to the same hemisphere. No participants showed the reversal of typical organisation. The findings are consistent with a statistical view of functional asymmetry, in which hemispheric biases for verbal and visual functions reflect probabilities relating to independent causal sources.

Functional differences between the two cerebral hemispheres are among the most replicated findings in all of neuropsychology. Typically, the most crucial areas involved in language production are found in the left hemisphere, while the right hemisphere is more specialised for visuospatial functions, though imaging studies of language emphasise that laterality is more a matter of degree rather than absolute division (Pujol, Deus, Losilla, & Capdevila, 1999). The origin of this division of labour, termed complementary specialisation, remains unclear.

Two accounts of complementary specialisation may be distinguished. The first theory (the dependent biases hypothesis) proposes that the localisation of language and visuospatial functions is causally related. According to this theory, a particular function becomes localised to one hemisphere, because the contralateral hemisphere has already taken responsibility for the other. A number of potential mechanisms have been proposed for this causal effect (e.g., Hellige, 1990; Kosslyn, 1987). One account comes from Cook (1984) and the notion of callosal inhibition. According to this theory, homotopic areas between the hemispheres inhibit one another, as do adjacent areas within the same hemisphere. When an area in one hemisphere is inhibited, the adjacent area in the same hemisphere is activated, leading to inhibition of the homotopic area in the contralateral hemisphere. This cycle may result in different hemispheres becoming dominant for functions performed in closely contiguous neural areas. In the case of language and visuospatial abilities, a brain region specialised for information processing may lead these two complementary functions to be divided between the hemispheres.

The second view (independent biases hypothesis) contrasts with the first, maintaining that complementary specialisation is a statistical rather than causal phenomenon (Bryden, Hécaen, & DeAgostini, 1983). According to this view, there is population bias for language to be lateralised to the left hemisphere and visuospatial skills to the right, but these biases reflect probabilities relating to independent causal sources, which may be genetic, biological or environmental in origin (or a combination of these). Primary evidence for this theory comes from studies that have examined verbal and visuospatial deficits among patients with a unilateral brain lesion. Most patients demonstrate verbal impairments following a left-hemisphere lesion and visuospatial impairments following a right-hemisphere lesion. However, a small number of patients show dual impairment following unilateral damage (Bryden et al., 1983), suggesting that functional division is not an obligatory pattern of cerebral organisation.

To date, investigations in this area have been limited for two reasons. First, for many years there existed only indirect techniques for determining laterality, such as through examinations of handedness, dichotic listening and visual-half fields (for a review, see Hellige, 1990). While these behavioural methods are known to be associated with cerebral lateralisation, correlation with actual neural organisation is far from perfect (Bishop, 1990). It is also difficult to draw firm conclusions regarding lateralisation from reports of patients with ‘crossed-aphasia’ (aphasia after right-hemisphere damage) or ‘crossed non-aphasia’ (left-hemisphere damage without aphasia but with visuospatial impairments), because of the possibility that cortical reorganisation had taken place post-insult. Second, studies employing more direct methodologies, such as the Wada technique and functional Magnetic Resonance Imaging (fMRI), have tended to investigate language and visuospatial skills in isolation (e.g., Postle, Stern, Rosen, & Corkin, 2000; Pujol et al., 1999), while those investigations that have studied laterality for both abilities have tended to include relatively small sample sizes (e.g., Gur et al., 2000), making it difficult to extrapolate findings to the wider population. Studies that use direct techniques to determine the cerebral laterality of both verbal and visuospatial abilities with a large number of participants will provide important data toward the goal of understanding the origins of complementary specialisation in humans.

One approach that may assist in this goal is functional transcranial Doppler sonography (fTCD). This non-invasive technique uses ultrasound to measure event-related changes in blood-flow velocity in the middle cerebral artery (MCA) serving each hemisphere. As with fMRI, fTCD works under the premise that increases in neural activity leads to greater glucose and oxygen consumption that must be replenished via enhanced blood flow to the area (Lohmann et al., 2006). By comparing the event-related changes in blood-flow velocity through the two MCAs, it is possible to determine the lateralisation of specific cognitive functions. A common experimental paradigm used to determine language laterality with fTCD is the word generation task, in which individuals are required to silently generate words that begin with a given letter. This method gives high correlations with existing ‘gold standard’ measures of language laterality, such as fMRI (Deppe et al., 2000) and the Wada test (Knecht et al., 1998), but is considerably quicker and less expensive to undertake.

The advent of fTCD as a neuroimaging tool has led to numerous attempts to map the relation between verbal and visuospatial lateralisation (Bulla-Hellwig, Vollmer, Gotzen, Skreczek and Hartje, 1996; Hartje, Ringelstein, Kistinger, Fabianek, & Willmes, 1994). However, many of these studies pre-dated the development of techniques that minimised recording artefacts (e.g., activity from the heart rate cycle), and consequently there were difficulties obtaining reliable measurements of hemispheric activation. Employing an analytic method devised by Deppe, Knecht, Henningsen, and Ringelstein (1997), Flöel et al. (2001) examined activation for spatial attention in healthy adults with either typical or atypical hemispheric dominance for language. Spatial attention was found to lateralise to the right hemisphere in the majority of people with left-hemisphere language, while there was a complementary hemispheric reversal of function in those individuals with the atypical pattern of language dominance. Importantly, in a small number of cases, these functions were found to lateralise to the same hemisphere (Flöel, Buyx, Breitenstein, Lohmann, & Knecht, 2005), indicating that hemispheric division of function, at least for these skills, is not observed in every individual.

The current study sought to extend the findings of Flöel et al. (2001, 2005), by investigating lateralisation for language and for another aspect of visuospatial ability, spatial memory. Like spatial attention, spatial memory is known to be typically subserved by a right-hemisphere dominant network of frontal and parietal sites (for a review see, Awh & Jonides, 2001). However, it remains unclear as to how spatial attention and spatial memory relate to each other. For example, these mechanisms may develop on a different timescale, with the storage function of spatial memory a ‘downstream’ consequence of early sensory attention. This may lead the cortical network involved in these functions to develop under different organisational influences.

The aim of the current study was to examine complementary specialisation for language and spatial memory. A dependent biases theory predicts that a division in hemispheric specialisation of function would be observed in *all* healthy adults. At the population level, this would lead to a negative association between language and visuospatial laterality, where the majority of individuals with left-hemisphere lateralised language would have right-hemisphere lateralised language, while a small proportion of cases would show the reverse. An independent biases theory also predicts that the bulk of the population would have language and visuospatial skills lateralised to the left- and right-hemisphere respectively. However, because the functional dissociation is a reflection of two independent biases (and not an obligatory mechanism), there will be a small proportion of individuals who do not show one bias or the other, leading to a situation where both functions are lateralised to the same hemisphere. In even rarer cases, both biases would not be observed, leading to the reversal of the most typical pattern of cerebral organisation. Flöel et al. (2005) found that a division of function for spatial attention and language is not observed in all individuals, providing evidence for the independent biases theory of complementary specialisation. However, no study has used imaging techniques to examine lateralisation for language and spatial memory in a large participant sample.

## 1 Method

### 1.1 Participants

A total of 75 adults (48 females and 27 males), who had English as their first language and who had no history of neurological disorder, were recruited for this study. All participants were staff and students of Oxford University and aged between 18 and 56 years (*M* = 23.54, SD = 7.08). To maximise the chances of including individuals with an atypical pattern of lateralisation, non-right-handers were over-represented in the current sample. Handedness was measured using the Edinburgh Handedness Inventory (Oldfield, 1971), with scores of +40 or above denoting right-handedness, −40 or below denoting left handedness, and scores in between denoting ambidexterity. The sample included 45 right handed (28 females), 3 ambidextrous (2 females) and 27 left-handed individuals (18 females). Three further participants underwent testing but were excluded from the final sample either because a temporal bone window could not be found (*n* = 2) or because of noisy data (*n* = 1).

### 1.2 Apparatus and stimuli

A Doppler ultrasonography device (DWL Multidop T2: manufacturer, DWL Elektronische Systeme, Singen, Germany) was used to measure changes in blood-flow velocity through the right and left MCAs. Participants were fitted with a flexible head-set that held in place a 2-MHz transducer probe over each temporal skull window. Experimental tasks were presented on a Dell laptop computer and controlled by Presentation Software (Neurobehavioral Systems), which sent markers to the Multidop system to denote the start of each epoch.

### 1.3 Design and procedure

**Fig. 1:**
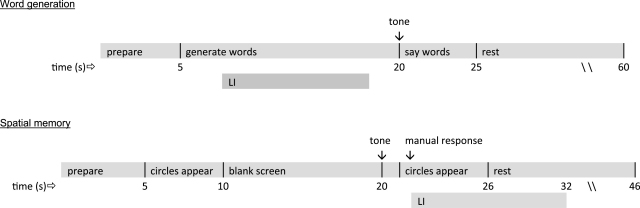
Timelines for the word generation and spatial memory tasks, also showing the periods of interest for Doppler recording.

Participants were seated in a quiet laboratory at Oxford University. A computer was placed on a table roughly 80 cm from the participant. Participants completed two computer-based tasks, each running for approximately 20 min. Fig. 1 presents the timelines for these tasks.

The first task involved the standard word generation task, a description of which is provided in full by Bishop, Watt and Papadatou-Pastou (2009). Briefly, participants were cued to attend to the computer screen. After 5 s, a letter from the alphabet appeared on the screen and participants were required to silently generate as many words that they could think of that began with that letter. Following a second cue 15 s later, participants were required to say out loud all of those words that they thought of during the silent generation period. A total of 23 trials were presented, with Q, X and Z the only letters omitted.

The second task assessed spatial memory and required participants to memorise the location of a number of circles. Participants were cued to attend to the computer screen, on which white (*n* = 17) and red circles (*n* = 9) appeared, on top of a black background. The circles, each 5 cm in diameter, were distributed evenly across the screen but not aligned in rows or columns (and therefore not conducive to verbal encoding). Participants were given instructions to memorise the location of the red circles, which were scattered randomly across the screen. The circles remained on the screen for 5 s and then disappeared, leaving a blank screen. After a period of 10 s, a tone sounded, and then the circle array appeared again, 1 s after that. In half of the 20 trials, the location of one of the red circles was swapped with one of the white circles. Participants sat with their hands on the table in front of them. They were asked to decide whether the red circles were the same or different as those that appeared in the initial screen, indicating their answer by raising the index finger on their left or right hand, respectively. Pilot testing found that this small motor movement made no discernable difference to overall activation levels. Furthermore, because there were 10 ‘same’ and 10 ‘different’ trials, any hemispheric activation caused by motor movement should be cancelled out across the 20 trials. Close to two-thirds of the participants (*n* = 47) had 90% accuracy or greater on this task, and no participant had less than 75% accuracy. ‘Same’ and ‘different’ trials were in the same random order for all participants.

Testing procedure differed slightly between participants. Just under one-quarter of the participants (*n* = 17, 22.7%; 13 females and four males; 14 right handed and three non-right handed) had the tasks split over two testing sessions, roughly two months apart. All of these participants received the word generation task in the first session and the spatial memory task in the second session. The remaining participants received both tasks in the same session (*n* = 58). The order of task presentation was counterbalanced, so that roughly half of the participants (*n* = 31; 53.4%; 16 right handed and 15 non-right-handed) received the word generation task followed by the spatial memory task, while the remainder (*n* = 27; 46.6%; 15 right-handed and 12 non-right handed) were administered the tasks in the reverse order.

### 1.4 Data analysis

Data were processed offline using the Autoedit function of the Average program version 1.85 (Deppe et al., 1997). This procedure downsamples the blood-flow envelope from each probe at a rate of 25 Hz, adds a channel corresponding to the heart beat, normalises the left and right cerebral blood-flow velocity curve to a mean of 100%, and removes heart beat activity, using the heart cycle integration described by Deppe et al. (1997). Epochs were set to begin 12 s before the cueing tone in both tasks, and to end at 30 s for the word generation task and 34 s for the spatial memory task. Data were subjected to an artefact rejection procedure, where epochs with unusually high or low levels of activity were removed. The baseline value was calculated as the mean velocity in the 12 s pre-cueing interval (*V*_pre.mean_). The relative changes in cerebral blood flow (d*V*) were calculated using the formula: d*V* = [*V*(*t*) − *V*_pre.mean_] × 100/*V*, where *V*(*t*) is the cerebral blood-flow volume over time.

A fTCD laterality index (LI) was calculated using the formula:

$$\text{L}{\text{I}}_{\text{fTCD}}=\frac{1}{t_{\text{int}}}\int_{t_{\text{max}}-0.5t_{\text{int}}}^{t_{\text{max}}+0.5t_{\text{int}}}\Delta V(t)\text{d}t$$

Here, Δ*V*(*t*) is the difference between the relative velocity changes of the left and right MCAs (i.e., left minus right) and *t*_max_ represents the latency of the absolute maximum of Δ*V*(*t*) during the predefined periods of greatest activation. Our own pilot testing found the periods of greatest activation to be the silent generation phase of the word generation task (8–18 s after the start of each trial) and the recognition phase of the spatial memory task (22–32 s after the start of each trial). A time period of 2 s was chosen for integration (*t*_int_).

The LI denoted the direction of laterality, with a positive index indicating greater left than right-hemisphere activation and a negative index signifying the reverse. More extreme LIs (i.e., strongly positive or negative) indicate a greater degree of lateralisation (Knecht et al., 2002). Because the LI for each task is a mean of a series of epochs (i.e., word generation maximum = 23; spatial memory maximum = 20), it was possible to determine whether this measure significantly differed from zero. A 95% confidence interval was computed around the each participant's LI on each task of each participant. If confidence intervals overlapped with zero, the participants were deemed to have bilateral activation.

## 2 Results

**Fig. 2:**
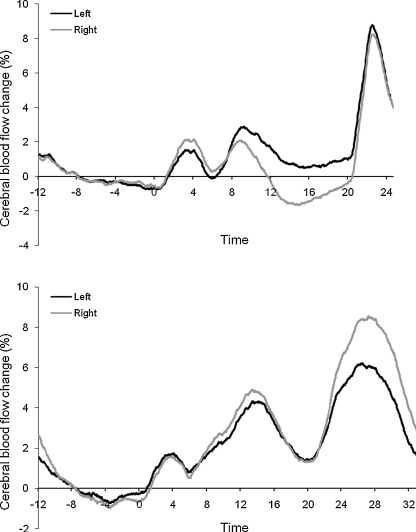
Average activation across accepted epochs for the left (black) and right (grey) middle cerebral artery. Activation for the language task is in the top panel, while the bottom panel shows activation for the spatial memory task.

All participants had at least 18 accepted epochs on the word generation task (*M* = 22.73; SD = .93) and 15 accepted epochs on the spatial memory task (*M* = 18.88; SD = 1.1). Fig. 2 shows the average activation from the left and right probes across all participants. A paired *t*-test found a significant difference between the mean LI for the word generation (*M* = 2.08, SD = 2.51) and spatial memory tasks (*M* = −2.36, SD = 2.39), *t*(74) = 13.01, *p* < .001, indicating that the two tasks successfully tapped left- and right-hemisphere activation, respectively.

Analyses then focused on determining whether there were fTCD differences between those who were tested over one or two sessions. The LIs of the 17 individuals tested over two sessions were compared with an equal number of participants who had their testing conducted in one session. The two groups were individually matched for sex (13 females and four males) and handedness (14 right handed and three non-right handed). There was no significant difference between the two groups in LI for the word generation (one session: *M* = 2.61, SD = 1.9; two sessions: *M* = 3.07, SD = 1.48; *p* = .44) and spatial memory tasks (one session: *M* = −2.57, SD = 2.17; two sessions: *M* = −1.83, SD = 2.57; *p* = .36). Among the 58 participants who were administered both tasks in one testing session, there was no effect of order of presentation for the word generation (word generation first: *M* = 1.84, SD = 2.56; word generation second: *M* = 1.73, SD = 2.85), *t*(56) = .67, *p* = .51, and spatial memory tasks (spatial memory second: *M* = −2.71, SD = 2.39; spatial memory first: *M* = −2.3, SD = 2.31), *t*(56) = .16, *p* = .87.

Split half reliabilities for each task were calculated by computing the LI values for the odd and even epochs and then correlating these. Strong positive correlations were found for both the word generation (*r* = .61) and spatial memory task (*r* = .59), which were both highly significant (*p* < .001).

Separate univariate ANOVAs examined the effect of sex (male vs. female) and handedness (right handedness vs. non-right handedness) on the LI for the two tasks. For the word generation task, there was a main effect for handedness, *F*(1,71) = 4.98, *p* < .05, but no effect for sex, *F*(1,71) = 2.67, *p* = .11, nor an interaction between the two variables, *F*(1,71) = .1, *p* = .72. No effects were found for the spatial memory task: handedness, *F*(1,71) = .82, *p* = .37; sex, *F*(1,71) = .7, *p* = .4; handedness x sex, *F*(1,71) = .02, *p* = .86.

Chi-square was used to examine the relationship between handedness (right handedness vs. non-right handedness) and hemispheric lateralisation. There was a trend for a greater proportion of non-right handed participants to show bilateral or right-hemisphere lateralised language function, relative to right-handed participants, *χ*^2^ = 4.35, df = 2, *p* = .11 (see Table 1). In contrast, the relation between handedness and lateralisation for spatial memory, presented in Table 2, was non-significant, *χ*^2^ = .09, df = 2, *p* = .96.

**Fig. 3:**
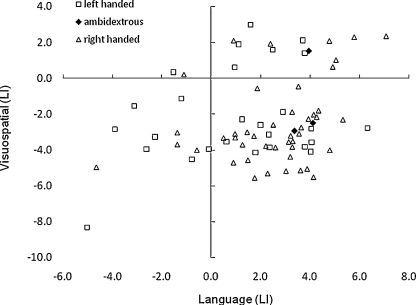
Laterality for language and spatial memory in right-handed, left-handed and ambidextrous participants. A positive index indicates greater left than right-hemisphere activation, with a negative index signifying the reverse. More extreme scores (i.e., strongly positive or negative) indicate a greater degree of laterality.

Analyses then examined the association between lateralisation for language production and spatial memory. Fig. 3 shows that the majority of participants (*n* = 48; 64%) had a positive index for language and negative index for spatial memory, while only two participants (2.7%) showed the reverse. There was a positive correlation of moderate strength between the two functions, *r* = .27 (*p* < .05).

Table 3 presents data on the lateralisation of these functions. Over half of the participants (53.3%) showed the ‘typical’ pattern of organisation with language and spatial memory lateralised to the left- and right-hemispheres, respectively. A further 16 participants (20.4%) had either left-lateralised language or right-lateralised spatial memory, and bilateral distribution of the other function. Sixteen participants had these functions lateralised to the same hemisphere, while no participant showed the reverse pattern of typical organisation. The association between laterality for language and for spatial memory was not statistically significant on Chi-square, *χ*^2^ = 3.38, df = 4, *p* = .49.

## 3 Discussion

The findings provide clear evidence for the independent biases theory of complementary specialisation. A dependent biases theory would predict that laterality indices for language and spatial memory would be inversely correlated in the population. The data presented in Fig. 3 and Table 3 shows that this was not the case. At the population level, there is a bias for language to be represented in the left hemisphere, and for visuospatial memory to be represented in the right hemisphere, but these biases are independent. When viewed categorically, it was common for participants to have left-lateralised language and right-lateralised visuospatial ability, but the reverse pattern was rare. Indeed, among those with language lateralised to the right-hemisphere (*n* = 10), only two participants had a positive LI for spatial memory (indicating greater left- than right-hemisphere activation), and neither of these significantly differed from zero. In comparison, there were a number of participants who had both functions lateralised to either the left- (*n* = 8) or right-hemisphere (*n* = 8). These data are congruent with the findings of Flöel et al. (2005), and suggest that organisational influences upon spatial attention and spatial memory, at least at the macro-level (i.e., lateralisation), may be similar. Taken together, these findings are consistent with the notion that the typical organisation of verbal and visuospatial functions reflects influences from independent sources.

The current data can also be used to test a specific prediction from the Right Shift Theory (Annett & Alexander, 1996). According to this theory, the majority of the population have a single or double copy of an allele that corresponds to a “right shift factor”, biasing language laterality to the left and handedness to the right. Individuals who do not have this factor will have laterality determined by chance, and this chance factor will operate independently for all lateralised functions. Thus this theory adopts a version of the independent biases hypothesis just for the subset of the population without the right shift factor, who will have a 50% chance of left- or right-hemispheric laterality for any given function. According to the theory, people with bilateral or right-hemisphere speech lack the right-shift factor; therefore, their visuospatial skills should be equally likely to be lateralised to the right or left. As can be seen from Table 3, this was not found; instead 16 of the 19 people with bilateral or right-hemisphere language had the standard pattern of right-hemisphere visuospatial skills. This proportion differs significantly from 50% (*χ*^2^ = 12.2, df = 1, *p* < .001).

However, our findings are compatible with studies of patients with unilateral brain lesions (Annett and Alexander, 1996; Trojano, Balbi, Russon, & Elefante, 1994). Bryden et al. (1983) have provided perhaps the most comprehensive study in this area, administering a full neuropsychological assessment to 270 patients with a unilateral brain lesion. As with the current study, a minority of patients were found to have both verbal and visuospatial impairment following lesion, indicating that, in these individuals, both of these functions were lateralised to the same hemisphere. An even smaller percentage of patients showed a ‘crossed’ pattern of asymmetry, with aphasia associated with intact visuospatial abilities following a right-hemisphere lesion, and the reverse pattern following a left-hemisphere lesion. The data from the current study suggest that the findings of Bryden et al. do not merely reflect cerebral reorganisation following lesion, but rather that these patients may have had an atypical pattern of lateralisation prior to their brain insult, similar to that observed in a minority of participants in the current sample.

No participant in the current sample had a history of neurological disorder and all participants had completed or were currently attending University education. Atypical language lateralisation has been implicated in a number of neurodevelopmental disorders, including schizophrenia (Sommer, Ramsey, & Kahn, 2001), specific language impairment (Whitehouse & Bishop, 2008) and dyslexia (Habib, 2000). However, atypical language dominance is not necessarily associated with a behavioural cost. Knecht et al. (2001) found no difference on a range of behavioural measures, including mastery of foreign languages, artistic talent, and verbal fluency, between participants with left (*n* = 264), bilateral (*n* = 31) and right-hemisphere (*n* = 31) language representation. The current findings extend those of Knecht et al. in showing that having both verbal and visuospatial skills lateralised to the same hemisphere is not necessarily detrimental to academic achievement (though the current participants were not assessed for mild forms of dyslexia). While the limited spatial resolution of fTCD does not allow us to draw further conclusions regarding the cortical distribution of these functions, fMRI studies indicate that there may also be intrahemispheric variability between individuals with the same interhemispheric pattern of lateralisation (D’Esposito et al., 1998).

An additional finding of the current study was that handedness was associated with cerebral laterality for language, but not for spatial memory. A large number of studies using a range of neuroimaging techniques (Szaflarski et al., 2002), including fTCD (Knecht et al., 2000), have identified that atypical cerebral dominance for language is more common among non-right handed individuals relative to right-handed individuals. The current study corroborates these data. The relation between handedness and lateralisation for spatial abilities has received considerably less research attention. The existing literature has been summarised by Vogel, Bowers, and Vogel (2003) in a meta-analysis that examined cerebral laterality for spatial ability in the context of a number of potential moderator variables, including handedness. While right-handed individuals were found to have a strong right-hemisphere bias for these abilities, there was no such association among left-handed individuals. These data clearly differ from those of the current study, in which right- and non-right handed individuals showed a similarly strong bias towards right-hemisphere processing of spatial memory (Table 2). The discrepancy in findings may relate to the methodology of the studies included in the meta-analysis, the majority of which used behavioural measures for determining cerebral laterality for spatial ability. This highlights the caution that must be taken when interpreting behavioural measures of cerebral laterality.

Considerable research has investigated what may cause the left-hemisphere bias for language processing. A range of factors has been proposed including genes (Annett, 1985; McManus, 2002), levels of foetal testosterone (Geschwind & Galaburda, 1985), and asymmetry in the intrauterine environment (Previc, 1991). At the neurobiological level, there is some evidence of microscopic differences in organisation between left and right hemispheres that may facilitate different types of neural computation (Buxhoeveden et al., 2001). Our data cannot speak directly to the issue of whether genetic or environmental influences are more important, but they do provide a challenge to theories that explain both left-hemisphere specialisation for language and right-hemisphere specialisation for visuospatial functioning in terms of a common mechanism.

## Figures and Tables

**Table 1:** Cerebral lateralisation for language, shown as a function of handedness. The parentheses denote the proportion of participants with that pattern of lateralisation within each handedness category.

Language	Handedness	
	Non-right	Right
Left	20 (66.7)	36 (80)
Bilateral	3 (10)	6 (13.3)
Right	7 (23.3)	3 (6.7)

**Table 2:** Cerebral lateralisation for spatial memory, shown as a function of handedness. The parentheses denote the proportion of participants with that pattern of lateralisation within each handedness category.

Language	Handedness	
	Non-right	Right
Left	4 (13.3)	5 (11.1)
Bilateral	4 (13.3)	6 (13.3)
Right	22 (73.3)	34 (75.6)

**Table 3:** Crosstabulation showing the participants’ cerebral lateralisation for language and spatial memory, based upon LIs and 95% confidence intervals. Participant numbers are presented, with proportion of total participants in parentheses.

Spatial memory	Language			
	Left	Bilateral	Right	Total
Left	8 (10.7)	1 (1.3)	0 (–)	9 (12)
Bilateral	8 (10.7)	0 (–)	2 (2.7)	10 (13.3)
Right	40 (53.3)	8 (10.7)	8 (10.7)	56 (74.7)
Total	56 (74.7)	9 (12)	10 (13.3)	75 (100)
