# Corrigendum to “Hemispheric division of function is the result of independent probabilistic biases” [Neuropsychologia 47 (2009) 1938–1943]

**DOI:** 10.1016/j.neuropsychologia.2012.05.029

**Published:** 2012-07

**Authors:** Andrew J.O. Whitehouse, Dorothy V.M. Bishop

**Affiliations:** a Telethon Institute for Child Health Research, Centre for Child Health Research, The University of Western Australia, PO Box 855, West Perth, Western Australia 6872, Australia; b Department of Experimental Psychology, University of Oxford, South Parks Road, Oxford OX1 3UD, United Kingdom

The authors have detected a typographical error in Table 2 of this paper. The error is in the top left hand corner of the table where the word ‘language’ is written. As stated in Table 2 caption, this table refers to cerebral lateralisation for spatial memory and not language. Thus, rather than ‘language’, the label should read ‘spatial memory’. This typographical error has no bearing on the results or conclusions of the study, which remain unchanged.

## Figures and Tables

**Table 2:** Cerebral lateralisation for spatial memory, shown as a function of handedness. The parentheses denote the proportion of participants with that pattern of lateralization within each handedness category.

Spatial memory	Handedness	
	Non-right	Right
Left	4(13.3)	5(11.1)
Bilateral	4(13.3)	6(13.3)
Right	22(73.3)	34(75.6)

